# Longitudinal evaluation of bone mass in adolescents and young adults with juvenile idiopathic arthritis: the role of bone mass determinants in a large cohort of patients

**DOI:** 10.1186/1546-0096-9-S1-P159

**Published:** 2011-09-14

**Authors:** Falcini Fernanda, Stagi Stefano, Cavalli Loredana, Masi Laura, Capannini Serena, Ceri Lorenzo, Matucci Cerinic Marco, Brandi Maria Luisa

**Affiliations:** 1 Dpt of Internal Medicine, Section of Rheumatology, Transition Clinic, University of Florence, Florence, Italy; 2 Pediatric Unit, Mugello’s Hospital, Borgo San Lorenzo, Florence, Italy; 3 Dpt of Internal Medicine, Endocrinology Unit, University of Florence, Florence, Italy

## Background

There are very few data evaluating prospectively the bone mass and quality determinants using dual energy X-ray absorptiometry (DXA), peripheral quantitative computed tomography (pQCT), and ultrasound (US) in JIA.

## Aim

To evaluate bone mass and quality determinants, and to identify the main predictors of reduced Bone Mineral Density (BMD) and bone quality using these techniques in JIA.

## Methods

One hundred and fifty-one patients (median 15.6, range 9.7 to 35.2 years; 101 oligoarticular, 30 polyarticular, 15 systemic, and 15 enthesitis-related–arthritis onset [ERA]) were evaluated. Of these, fifty-nine consecutive patients were followed-up with a second evaluation. The data obtained were compared with 80 ages- and sex- matched healthy subjects.

## Results

At the first evaluation, JIA patients showed a reduced spine aBMD SDS value in comparison to controls (p < 0.005). JIA patients showed also significant musculoskeletal deficits, with lower levels of TrabBMD (p < 0.0001), muscle CSA (p < 0.005), SSIp (p < 0.05), and significantly increased levels of fat CSA than controls (p < 0.0001). Finally, JIA patients presented a significantly reduced AD-SoS (p < 0.001), and QUS z-score (p < 0.005). These data were confirmed also in longitudinal evaluation. However, evaluating different treatments, we showed a significant negative correlation among aBMD value (p < 0.005), TrabBMD (p < 0.005), AD-SoS (p < 0.005), and systemic corticosteroids exposure or intra-articular corticosteroids injections, and a positive correlation among TNF-alpha-blocking agents and aBMD (p < 0.005), TrabBMD (p < 0.005), and AD-SoS (p < 0.005).

## Conclusions

Pts with JIA have a low bone mass and quality in comparison to controls, and do not reach the normal condition over time despite the current more effective drugs.

